# Postrenal Acute Renal Failure Due to Giant Fecaloma-related Bilateral Hydronephrosis: A Case Report and Brief Literature Review

**DOI:** 10.7759/cureus.7815

**Published:** 2020-04-24

**Authors:** Deniz Noyan Ozlu, Kamil Gokhan Seker, Yurdagul Cetin Seker, Ahmet Haciislamoglu, Nadir Kalfazade

**Affiliations:** 1 Urology, Bakirkoy Dr. Sadi Konuk Training and Research Hospital, Istanbul, TUR; 2 Emergency Medicine, Okmeydanı Training and Research Hospital, Istanbul, TUR

**Keywords:** fecaloma, renal failure, obstructive uropathy, hydronephrosis

## Abstract

An 88-year-old woman presented to the emergency department with abdominal distention, fever, and constipation of about a week's duration. Laboratory tests showed impaired kidney function tests and fluid electrolyte values. Bilateral hydroureteronephrosis was observed on non-contrasted abdominal CT. Imaging revealed no intrinsic urological pathology (ureteral stones, etc.) that could lead to obstruction in the urinary system; however, excessively dilated and feces-loaded rectum and colon were observed. The patient was treated with conservative methods. Unfortunately, she passed away due to general condition disorder.

## Introduction

Fecaloma is formed due to the presence of organized, hard fecal residue in the colon and rectum for a long time. Sigmoid colon and rectum are the two regions where fecaloma is frequently seen. Significant complications may occur in the gastrointestinal system due to fecaloma formation. Due to the local pressurizing effect of fecaloma (pelvic mass effect), severe morbidity such as urinary system obstruction and rupture of colon or bladder may occur, and sometimes they can be mortal [1]. Although conservative methods such as the use of enemas, laxatives, and rectal evacuation are among the treatment options to relieve the fecal effect, surgical intervention is another option when these methods fail [2]. Rare cases of fecaloma-related urinary system obstruction and renal failure have also been reported [1,3,4].

In this report, we present a case of postrenal acute renal failure due to giant fecaloma-induced bilateral hydronephrosis in a female patient admitted to the emergency department with constipation. We also present a brief review of the relevant literature.

## Case presentation

An 88-year-old female patient was admitted to the emergency department with symptoms of constipation, weakness, and fever for about a week. Her past medical history was remarkable for hypertension, congestive heart failure, diabetes mellitus, constipation, and recurrent urinary tract infection. She also had a history of regular use of acetylsalicylic acid, amlodipine, furosemide, and pioglitazone. On initial evaluation, the patient had a blood pressure of 140/90 mmHg, a pulse rate of 111 beats/min, and a body temperature of 37.9 ^o^C. Her urine output was decreased (about 300 cc in the last 24 hours). Physical examination revealed abdominal swelling and sensitivity on lower abdominal quadrants. Bowel sounds were weak, and the rectum was full of fecaloid on digital rectal examination.

**Figure 1:**
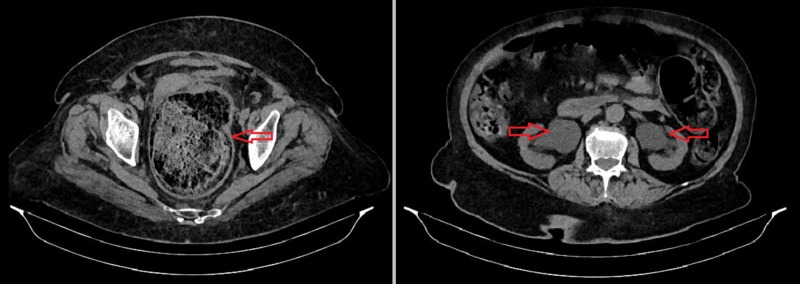
Non-contrast CT taken at admission Dilated and fecaloid-filled rectum and colon (left, red arrow) and bilateral hydroureteronephrosis in the urinary system (right, red arrows) can be seen CT: computed tomography

Among inflammation parameters, white blood cell (WBC) count was 15,100 cells/mcL (normal: 4,500-10,000 cells/mcL), and C-reactive protein (CRP) was 29 mg/L (normal: 0-5 mg/L) . Urine analysis was positive for WBCs (too numerous to count), red blood cells (RBCs, 15-25 per high power field), and bacteria (many). Creatinine increased to 3.49 mg/dL (normal: 0.6-1.1 mg/dL), and there was electrolyte imbalance in the biochemical analysis as her serum sodium was 110 mEq/L (normal: 135-145 mEq/L) and potassium was 5.67 mEq/L (normal: 3.3-5.1 mEq/L). Non-contrast CT of the abdomen showed dilated and fecaloid-filled rectum and colon, and bilateral hydroureteronephrosis in the urinary system (Figure 1). It was observed that the bladder was displaced anteriorly by the rectum filled with fecaloid.

**Figure 2:**
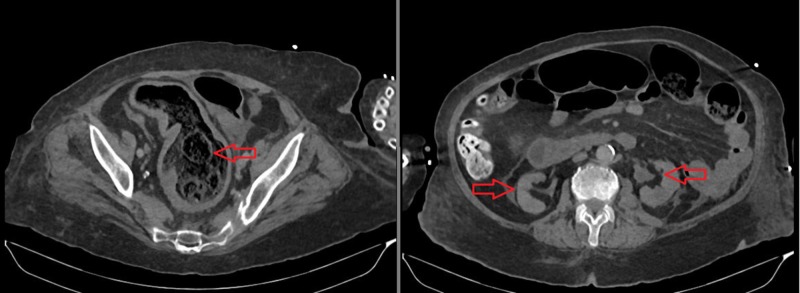
Non-contrast CT taken on day two of the admission After rectosigmoidal lavage, a decrease in the amount of fecaloid in the rectum (left, red arrow) and regression in the grade of hydroureteronephrosis (right, red arrows) can be seen CT: computed tomography

**Figure 3:**
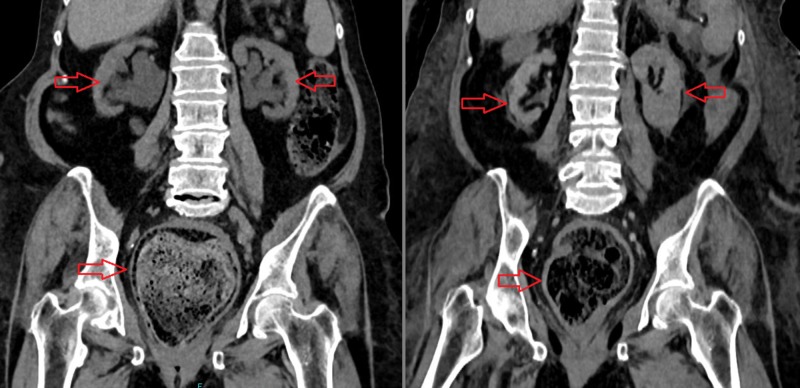
Non-contrast CT scan - coronal view At the time of admission (left): dilated and fecaloid-filled rectum and colon and bilateral hydroureteronephrosis in the urinary system (red arrows) can be seen. Two days later (right): after rectosigmoidal lavage, a decrease in the amount of fecaloid in the rectum and regression in the degree of hydroureteronephrosis (red arrows) can be observed CT: computed tomography

Her post-void residual of urine volume was about 20 cc. A Urethral Foley catheter was inserted into the patient and intravenous ceftriaxone (2 g/day) plus normal saline treatment were started. After general surgery and urology consultations, intensive rectosigmoidal lavage was applied through sodium picosulfate, rectal tube, and manual fecal extraction. Ureteral DJ stent placement was not planned due to the risk of carrying an ascending infection. Urine output increased right after rectosigmoidal lavage (about 200 cc/hour). A non-contrast abdominal CT, which was taken two days after the admission, revealed a decrease in the amount of fecaloid in the rectum and regression in the grade of hydroureteronephrosis (Figure 2). The serum creatinine level decreased to 1.39 mg/dL on day two. After lavage, a rapid increase in diuresis and a decrease in creatinine levels supported the diagnosis of postrenal acute renal failure. Regression in the grade of the hydroureteronephrosis and decrease in the amount of fecaloid in the rectum can be seen in Figure 3. Although fecaloma was extracted in the patient’s follow-ups, she passed away due to the degradation in her general condition and fluid electrolyte disorder a week after her admission.

## Discussion

Fecalomas are common in Hirschsprung's disease, Chagas' disease, patients with spinal cord injury, behavioral abnormality, and elderly patients with chronic constipation [3]. The risk factors in our case were advanced age and a history of recurrent constipation. In terms of the frequency of the disease, there is no significant difference in the ratio of women and men [4]. Usual fecaloma-related complications are intestinal obstruction, colonic ulceration, and stercoral perforation [4]. The urinary system can also be rarely affected due to the local pelvic mass effect. In the urinary system, urinary tract infection, hydronephrosis, and even bladder rupture may develop due to compression of the hard fecaloid onto the bladder [5]. The most common level of obstruction is the urethra or urethral-vesical junction. The mechanism of urinary retention caused by fecal impaction is believed to be a significant elevation of the floor of the bladder and posterior urethra with resultant obstruction of the bladder outlet [4]. Thus, intravesical ureters may be pressurized, and unilateral or bilateral hydronephrosis may occur [4,6].

As in our case, the local compression effect of fecaloma should be considered if there is deterioration in the renal function tests and obstructive uropathy findings, especially in patients in the geriatric age group who present to the emergency department with constipation. It should be considered as a cause of urinary tract obstruction and recurrent urinary tract infections. Fecalomas can also be confused with malignancies due to local pressure.

In the literature, urinary system complications of fecaloma have been presented in the form of several case reports. In 2016, Serrano Falcon et al. conducted a systematic review in which the complications of fecalomas were classified; they detected 9.76% of urological complications in 280 cases. These complications were obstructive uropathies (27 patients, 8.5%), and bladder complications (four patients, 1.26%) [7]. As far as we know, the first case of constipation-related hydroureteronephrosis was reported in 1954 by Ney and Hyman [8]. Claffey et al. have presented a case of bilateral hydronephrosis in a 71-year-old patient, which developed as a result of barium accumulation in the sigmoid colon and caused fecaloma after imaging with barium [9]. It was reported that hydronephrosis regressed after the removal of the fecaloma by rectal lavage without the need for an invasive procedure. Knobel et al. have reported giant fecaloma and related bilateral hydronephrosis in an 81-year-old female patient [10]. Yuri Iwata et al. have reported a fecaloma-related obstructive uropathy clinic in a 90-year-old female patient with a history of recurrent urinary tract infections and fever. Sodium picosulfate, intensive rectosigmoidal lavage, rectal tube, and manual fecal removal were applied. The clinical picture improved in the patient who was treated for urinary tract infection and whose bowel habits were controlled with lubiprostone [4]. Özer et al. have presented two cases where movement restriction due to knee arthroplasty operation and progressive muscular dystrophy (PMD) disease was shown to be the etiological factor. Both patients died during the follow-up [1].

McWilliams et al. have reported detecting a pelvic mass that filled the left lower quadrant, causing hydronephrosis and renal failure in a 74-year-old female patient who had a history of cerebrovascular event. Initially, it was thought that the patient had uterine leiomyoma or malignancy; however, after the removal of the fecaloma through the rectal lavage and enema, diuresis started, thereby providing regression in hydronephrosis and a decrease in creatinine values [11]. Özer et al have reported the case of a 73-year-old female patient who had knee arthroplasty; it was initially thought that she had uterine or ovarian tumoral mass-related bilateral hydronephrosis. But, ultimately, it was found that the actual pathology was due to the giant fecaloma. However, due to the failure of defecation via rectal lavage or enema, nephrostomy catheters were placed in the patient. In our case, exitus occurred due to fluid electrolyte and general condition disorder despite the discharge of the fecaloma [1].

Rectal emptying through rectal lavage, enema, and suppository treatments are generally applied in all patients. In the treatment of acute conditions, more invasive procedures should be considered in patients who do not respond to these interventions. Underlying pathology should be eliminated, and dietary changes and mobilization should be applied to prevent chronic constipation-related hydronephrosis [12,13]. Patients with acute renal failure usually recover quickly after fecaloma evacuation, and increased postobstructive diuresis is frequently seen. Although a good recovery was reported in most cases in the literature review made by Iwata in 2015, two deaths were reported due to constipation complications, and one of them was a sudden death after bladder perforation associated with the pelvic mass effect of massive feces [4].

## Conclusions

Although it is very rare, fecalomas causing pelvic mass effect should be considered as the cause of urinary system obstruction. The necessary interventions should urgently be made in these patients. Otherwise, a life-threatening condition may occur due to fluid electrolyte imbalance and deteriorated general conditions.
